# Self-report of ADHD shows limited agreement with objective markers of persistence and remittance

**DOI:** 10.1016/j.jpsychires.2016.07.020

**Published:** 2016-11

**Authors:** Ebba Du Rietz, Celeste H.M. Cheung, Gráinne McLoughlin, Daniel Brandeis, Tobias Banaschewski, Philip Asherson, Jonna Kuntsi

**Affiliations:** a King's College London, MRC Social, Genetic and Developmental Psychiatry Centre, Institute of Psychiatry, Psychology and Neuroscience, 16 De Crespigny Park, Denmark Hill, London SE5 8AF, United Kingdom; b Centre for Brain and Cognitive Development, Department of Psychological Sciences, Birkbeck, University of London, 32 Torrington Square, London WC1E 7JL, United Kingdom; c Department of Child and Adolescent Psychiatry and Psychotherapy, Central Institute of Mental Health, Medical Faculty Mannheim/ Heidelberg University, Square J5, 68159 Mannheim, Germany; d Department of Child and Adolescent Psychiatry, University of Zurich, Neumünsterallee 9, 8032 Zurich, Switzerland; e Center for Integrative Human Physiology, University of Zurich, Winterthurerstr. 190, 8057 Zurich, Switzerland; f Neuroscience Center Zurich, University of Zurich, Winterthurerstr. 190, Y17 H03, 8057 Zurich, Switzerland

**Keywords:** ADHD, Cognitive, EEG, Persistence, Self-report, Actigraph

## Abstract

**Objective:**

A controversial issue is whether self-report of symptoms and impairment is sufficient for diagnosis of attention-deficit/hyperactivity disorder (ADHD) in adolescents and adults in the absence of other informants, such as parents. The present study investigated how well self-report is reflected by cognitive-neurophysiological and actigraph measures, which we have previously shown to discriminate between ADHD persisters, remitters and controls using parent-report (Cheung et al., 2015; Brit J Psychiat http://dx.doi.org/10.1192/bjp.bp.114.145185).

**Method:**

Parent- and self-reported ADHD symptoms and impairment, together with cognitive, electroencephalogram (EEG) frequency, event-related potential (ERP) and actigraph measures were obtained from 108 adolescents and young adults with childhood ADHD and 167 controls.

**Results:**

Participants reported lower levels of ADHD symptoms and impairments than parents (p < 0.05) and the ADHD persistence rate based on self-report was low at 44%, compared to the persistence rate of 79% previously reported based on parent-report. Regression analyses showed that the objective measures distinguished poorly between ADHD persistent and remittent groups based on self-report, in contrast to findings based on parent-report (Cheung et al., 2015), although the measures differentiated well between ADHD persisters and controls. Correlation analyses revealed that self-reported impairment significantly correlated with fewer of the objective measures, despite parent- and self-reported symptoms showing similar correlations with the measures.

**Conclusions:**

The findings show that self-reported ADHD outcome is not as well reflected by cognitive-neurophysiological and movement correlates as we previously found for parent-reported ADHD.

## 1 Introduction

Attention-deficit/hyperactivity disorder (ADHD) is a childhood-onset neurodevelopmental disorder that frequently has long-term impact throughout the lifespan (National Institute of Health and Clinical Excellence; NICE, 2008). Childhood ADHD has an estimated prevalence of around 5.3% (95% CI: 5.01–5.56) world-wide (Polanczyk et al., 2007), and often persists into adulthood where the prevalence rate is 2.5% (95% CI: 2.1–3.1) (Simon et al., 2009). While parents and teachers are used as main sources for establishing diagnoses in children, self-report becomes increasingly important during diagnostic interviews in adolescence and young adulthood. There is, however, scarcity of research evaluating the validity of self-report compared to informant-report in establishing diagnosis of ADHD in adolescents and young adults.

Previous research suggests modest agreement between self- and parent-ratings of ADHD symptoms in adolescents and young adults (r = 0.16–0.30) (Barkley et al., 2002, Pierrehumbert et al., 2006, Wan Salwina et al., 2013). Young individuals tend to report their ADHD symptoms as less severe than their parents, which results in lower rates of ADHD persistence into adulthood based on self-report (Barkley et al., 2002, Kooij et al., 2008, Pierrehumbert et al., 2006). This suggests that follow-up studies that rely on self-report may estimate persistence of ADHD to be lower than studies using parent-report (Barkley et al., 2002, Wolraich et al., 2005). The exclusive reliance on adult self-report may have in part contributed to the low ADHD persistence rate of 5% recently reported by Moffitt et al. (2015), which is substantially lower than previous follow-up studies that have relied on both self- and parent-report and reported persistence rates between 15% and 35% (Biederman et al., 2010, Faraone et al., 2006). This discrepancy could also be explained by differences between population and clinical samples.

Overall, existing research is limited, yet suggestive evidence is emerging that self-report of ADHD may have lower validity than parent-report. Population-based and clinical ADHD studies have found that self-reported ADHD symptoms show weaker associations with poor school achievement in adolescence (Pierrehumbert et al., 2006) and major life events in young adulthood (Barkley et al., 2002), compared to parent-report. Furthermore, the estimated heritability of adolescent and adult ADHD based on self-reported symptoms (38–48%) (Larsson et al., 2013, Merwood et al., 2013) is lower than heritability estimates based on parent-reported symptoms (64–82%) (Cheung et al., 2015, Merwood et al., 2013), and clinically-diagnosed ADHD (88%) (Larsson et al., 2014), as defined by taking ADHD medication. The low heritability estimates for self-reported ADHD could be attributed to rater-bias effects introduced by using self-report, but is also likely due to the use of different informants to rate each twin in a pair rather than relying on a single informant (Brikell et al., 2015, Merwood et al., 2013).

While studies converge in suggesting that self-report of ADHD shows lower validity than parent-report, no studies have compared the validity of source informants using cognitive-neurophysiological and movement correlates of ADHD. Objective measures could be used to examine how well each informant report of ADHD is reflected by cognitive-neurophysiological and movement data.

We previously reported findings from a prospective study that successfully discriminated between ADHD persistent, remittent and control groups on cognitive-electrophysiological and actigraph measures (Cheung et al., 2015). The ADHD groups were based on parent-reports, given the relatively young age range of the sample (11–25 years), as literature suggests children with ADHD may be poor at judging their own problematic behavior (Hoza et al., 2002, Hoza et al., 2004). Preparation-vigilance processes (omission errors (OE), reaction time variability (RTV), contingent negative variation (CNV), delta activity), as well as IQ and actigraph count, were markers of remission in early adulthood. These processes distinguished between ADHD persisters and remitters, but not between ADHD remitters and controls.

We now examine ADHD persistence and remittance based on self-report in young adulthood using the same sample as our previous study (Cheung et al., 2015). The aim is to gain a better understanding of discrepancies between self- and parent-report and to investigate how well self-report is reflected by ADHD symptomology at the level of cognition, neurophysiology and movement. Given how ADHD is defined, we can examine inattentive, hyperactive and impulsive symptoms at an objective level of attention processes and fidgeting, although it is important to acknowledge that these are not regarded as gold-standard objective measures in the diagnostic process of ADHD and are limited to laboratory settings.

The main aims of the present study are to examine (i) whether self- and parent-report of ADHD differ in severity; (ii) how well the objective data discriminate between ADHD persisters, remitters and controls based on self-report of ADHD using DSM-IV criteria and (iii) the pattern of correlations between self-reported ADHD symptoms and impairments and the objective data.

Based on DSM-IV (The Diagnostic and Statistical Manual of Mental Disorders, 4th ed.), individuals are diagnosed with ADHD if they display at least six symptoms in either the inattentive or hyperactive-impulsive domains, and experience symptoms and impairment in at least two settings. In the revised DSM-5 criteria, individuals aged 17 or older only require the presence of five symptoms and the presence of symptoms in at least two settings, rather than impairments from symptoms in two settings. Thus, we run additional analyses to investigate whether the objective data discriminate better between ADHD groups, based on self-report, using the revised DSM-5 criteria of displaying at least 5 ADHD symptoms.

## 2 Method

### 2.1 Participants

The sample consists of 275 participants, followed-up on average 5.8 years (SD = 1.1) after initial assessments. At follow-up, participants were on average 18.0 years of age (age range: 11.1–25.9). 17 individuals were between 11 and 13 years, 79 individuals were between 14 and 16 years, 116 individuals were between 17 and 19 years and 63 individuals were 20 years and older. 108 participants had a diagnosis of DSM-IV combined type ADHD in childhood (9 sibling pairs, 90 singletons) and 167 were controls (74 sibling pairs, 19 singletons).

Participants with ADHD were initially recruited from ADHD clinics in south-east England (Kuntsi et al., 2010). Diagnosis of DSM-IV combined type ADHD was established using the Parental Account of Childhood symptoms (PACS), a semi-structured interview with high inter-rater reliability (Chen et al., 2008). Controls were recruited from schools in the same region and were age and sex matched with the clinical sample. All participants were aged between 6 and 17 at initial assessment. Exclusion criteria were: IQ < 70, autism, epilepsy, general learning difficulties, brain disorders and any genetic or medical disorder associated with externalizing behaviors that might mimic ADHD. The investigation was carried out in accordance with the latest version of the Declaration of Helsinki. The study design was reviewed by an appropriate ethical committee and informed consent of participants was obtained after the nature of the procedures had been fully explained.

At follow up, eight controls met ADHD criteria based on self- (n = 2) or parent- (n = 6) ratings on the Barkley Informant Rating Scale, and eight participants had missing self- or parent-ratings of impairments. These participants were excluded from analyses.

### 2.2 Procedure

Participants were scheduled for a follow-up clinical interview and cognitive-electroencephalogram (EEG) assessments at the research center where initial assessments took place. A 48-h ADHD medication-free period was required. The total length of the test session, including breaks, was approximately 4 h.

### 2.3 Measures

*The Diagnostic Interview for ADHD in adults (DIVA)* is a semi-structured interview evaluating the DSM-IV criteria for adult and childhood ADHD symptoms and impairment (Kooij and Francken, 2007). The DIVA was conducted by trained researchers with participants and parents separately.

*The Barkley's functional impairment scale (BFIS)* (Barkley and Murphy, 2006). This 10-item scale assesses levels of functional impairments associated with ADHD symptoms in five areas: family/relationship; work/education; social interaction; leisure activities and management of daily responsibilities. Each item ranged from 0 (never or rarely) to 3 (very often). Participants and parents both completed the questionnaire.

Participants were classified as ADHD persistent at follow-up based on DSM-IV criteria; if they scored ‘yes’ on ≥ 6 items in either the inattention or hyperactivity-impulsivity domains, and if they scored ≥2 on two or more areas of impairments.

*Barkley Informant Rating Scale* (Barkley and Murphy, 2006). This rating scale (based on DSM-IV items) was used to identify controls meeting ADHD diagnostic criteria at follow up. Each item ranged from 0 (never or rarely) to 3 (very often). Participants and parents both completed the questionnaire.

*IQ and digit span.* The vocabulary and block design subtests of the Wechsler Abbreviated Scale of Intelligence (WASI) were administered to derive an IQ estimate (Wechsler, 1999). The digit span subtest from the WISC-III (Wechsler, 1991) or the WAIS-III (Wechsler, 1997) was administered to participants aged below 16 and aged 16 or above, respectively, to obtain digit span forward (DSF) and backward (DSB). DSF requires participants to verbally repeat a sequence of digits in straightforward order, and measures short-term verbal memory. DSB requires participants to repeat digits in backward order, and measures verbal working memory.

*Actigraph measures of activity level*. Actigraph readings were taken during interviews and assessments. We previously showed that mean intensity and mean number of movements, obtained from the dominant ankle, reliably distinguished between ADHD probands and controls (ROC-AUC = 0.61–0.79) (Wood et al., 2009).

*The Fast Task* (Andreou et al., 2007). The baseline condition consists of 72 trials, which followed a standard warned four-choice RT task. Four empty circles (warning signals, arranged horizontally) first appeared for 8s, after which one of them (the target) was colored in. Participants were asked to press the response key corresponding to the target position. Following responses, the stimuli disappeared and a fixed inter-trial interval of 2.5s followed. Speed and accuracy were emphasized equally. If participants did not respond within 10s, the trial terminated. A comparison condition with a fast event rate (1s) and incentives followed the baseline condition. We used the RTV from the baseline condition, as this condition is more sensitive to ADHD (Kuntsi et al., 2013).

*The cued flanker Continuous Performance Task (CPT-OX)* (Doehnert et al., 2008, Valko et al., 2009). This CPT includes rare cued Go and NoGo conditions embedded in a vigilance task with frequent distractors to assess attention and inhibition. 400 letters are presented for 150 ms with a stimulus onset asynchrony of 1.65 s in a pseudo-randomized order. The cue letter O occurred with 20% probability (80 Cue stimuli), signaling a Go–NoGo task. Participants pressed a button as fast as possible every time the cue was followed directly by the letter X (O-X) target sequence (10% probability, 40 Go stimuli), but had to withhold responses to O-not-X sequences (NoGo trials, also 10%, 40 NoGo stimuli). RTV, commission errors (CE), OE; EEG frequency bands; and event-related potential (ERP) amplitude measures of CNV, cue-P3 and nogo-P3 were obtained.

### 2.4 EEG recording and processing

EEG was recorded from 62 channels DC-coupled recording system (extended 10–20 montage), with a 500 Hz sampling-rate, impedances kept under 10 kΩ and FCz as the reference electrode. The electro-oculograms (EOGs) were recorded from electrodes above and below the left eye and at the outer canthi.

The EEG data were analyzed using Brain Vision Analyzer (2.0) (Brain Products, Munich, Germany). After down-sampling the data to 256 Hz, the EEG data were re-referenced to the average and filtered offline with digitally band-pass (0.1–30 Hz, 24 dB/oct) Butterworth filters. Ocular artifacts were identified using Independent Component Analysis (ICA) (Jung et al., 2000). The extracted components were manually inspected and ocular artifacts were removed by back-projection of all but those components. Data with other artifacts exceeding ±100 μV in any channel were rejected. No baseline subtraction was applied in line with previous ERP analyses on this task (Doehnert et al., 2013, McLoughlin et al., 2011). All averages contained at least 20 sweeps.

### 2.5 ERP analyses

The CNVs were analyzed as mean amplitudes 1300–1650ms following cues over the central electrode (Cz). The cue-P3 had a parietal maximum and was defined as the most positive peak 250–600ms following cue trials at electrode Pz. The nogo-P3 was defined as the most positive peak 250–600ms following no-go trials at electrode Cz.

### 2.6 EEG frequency analyses

We estimated mean EEG power (μV^2^) by computing the mean activity of electrodes (F1—F8, Fz) in the delta (0.5–3 Hz), theta (4–7 Hz), alpha (7–12 Hz) and beta (12–30 Hz) bands using the Fast Fourier Transform (FFT). We analyzed the frontal location only, to be consistent with our previous analyses (Cheung et al., 2015).

### 2.7 Statistical analyses

We ran regression models with dummy variables to identify which measures showed an effect of group (ADHD persisters vs ADHD remitters vs controls), with controls as the reference group. Post-hoc *t*-tests were conducted to examine ADHD persistent-remittent differences. We explored the effect of sex by re-running analyses with females (n = 55) removed. We also re-ran the analyses using groups based on DSM-5 criteria of having five, rather than six, ADHD symptoms. Cohen's *d* effect sizes are presented with means, SDs and test statistics for the group analyses; 0.2 is considered a small effect, 0.5 a medium effect and 0.8 a large effect.

Pearson correlations were conducted on the objective measures to examine their associations with DIVA ADHD symptom scores and clinical impairment, within those with childhood ADHD, with age and gender included as covariates.

We ran additional analyses to investigate whether the combination of information from self- and parent-reports is better reflected by the objective measures compared to only using parent-report. We compared profiles of individuals with both self- and parent-reported ADHD (concordant group), individuals with only parent-reported ADHD (discordant group) and controls on the objective measures and reports of impairment. We did not examine individuals with only self-reported ADHD as this group of individuals was too small (n = 17).

We re-ran all analyses covarying for IQ to examine its potential effects. All cognitive and EEG measures were skewed and log-transformed to normal in STATA version 10 (StataCorp, College Station, TX). Genetic relatedness between the sibling pairs was controlled for by using the ‘robust cluster’ command in STATA.

## 3 Results

Based on self-reports of symptoms and impairment, 44% of individuals with childhood ADHD continued to meet DSM-IV levels of ADHD and were classified as ADHD persisters. As reported previously (Cheung et al., 2015), 79% of individuals were classified as ADHD persisters based on parent-report. Using DSM-5 symptom criteria, 47% of individuals were classified as ADHD persisters based on self-report, while the persistence rate remained the same for parent-report.

At follow up, based on self-report, ADHD persisters, remitters and controls did not differ in age, but there were significantly more males in the remitted group than the control group (Table 1). The follow-up duration was not significantly different between persistent and remittent groups (*z* = 0.31, *p* = 0.76).

Almost half (47%) of the participants were under medication treatment for ADHD at the time of the follow-up assessment. The proportion of participants on medication at follow up did not differ between persistent and remittent groups based on either self-report (χ = 1.46, p = 0.23) or parent-report (χ = 1.95, p = 0.16).

### 3.1 Do self-reports of ADHD symptoms and impairments differ in severity from parent-reports in individuals with a childhood diagnosis of ADHD?

The average number of self-reported inattentive symptoms (M = 5.82, SD = 0.23) was significantly lower (t (107) = 6.85, p < 0.001) than the number of parent-reported symptoms (M = 7.44, SD = 0.19). Self-reported hyperactive-impulsive symptoms (M = 4.86, SD = 0.23) was significantly lower (t (107) = 3.54, p < 0.001) than parent-reported hyperactive-impulsive symptoms (M = 5.84, SD = 0.25). Self-reported impairment was significantly lower (t (105) = 4.67, p < 0.001) for self-report (M = 11.01, SD = 5.73) than parent-report (M = 13.91, SD = 6.64). There was a significant correlation between self- and parent-reported ADHD symptoms (r = 0.37, p < 0.001), as well as impairments (r = 0.48, p < 0.001).

### 3.2 Which processes are impaired in the ADHD persistent group based on self-report?

ADHD persistent-control group differences were observed on all measures except for EEG delta, theta and beta activity, and movement count (p > 0.05). After controlling for IQ, there were no longer significant ADHD persistent-control differences on DSF, RTV (from CPT-OX) and alpha activity (Table 1). Controlling for IQ led to slight reductions in effect sizes for most variables; however, the effect size was still large for RTV from Fast Task (Table 1). When we re-ran the analyses excluding females, the pattern of findings did not change.

### 3.3 Which processes are impaired in the ADHD remittent group defined by self-report?

ADHD remittent-control group differences were observed on the same measures that distinguished ADHD persisters from controls, except for cue-P3. After controlling for IQ, ADHD remittent-control group differences remained only for RTV (Fast Task), CE and OE, and the effect sizes were reduced (Table 1).

When we re-ran the analyses with females removed a significant ADHD remittent-control difference in EEG beta activity emerged, and there were no longer significant differences on CNV and DSF between remitters and controls (p > 0.05). The results did not change for the remaining variables.

### 3.4 Which processes are markers of remission that distinguish between ADHD persistent and remittent groups defined by self-report?

A marker of remission refers to a measure that distinguishes ADHD remitters from persisters, but not from controls. In this study, ADHD persisters and remitters only significantly differed on RTV (Fast Task). However, as the measure also distinguished ADHD remitters from controls (p < 0.05) (Table 1), it does not fulfill the criteria as a marker of remission but rather represents an intermediate deficit in ADHD remitters. After controlling for IQ, the group ADHD persistent-remittent group difference remained significant for RTV (p < 0.001) and the effect size increased slightly (from d’ = 0.54 to d’ = 0.61). The pattern of results did not change when analyses excluded females.

### 3.5 How well do the objective data discriminate between ADHD groups based on DSM-5 diagnostic symptom criterion?

When ADHD status was based on self-reports using the DSM-5 symptom criterion of 5 rather than 6 symptoms, three individuals were re-classified as ADHD persisters, from being ADHD remitters according to DSM-IV. The group-based analyses based on DSM-5 criteria showed the same results as when groups were based on DSM-IV criteria, with the exceptions that significant ADHD remittent-control group differences emerged on the nogo-P3, intensity movement count and beta activity, and there was no longer a significant ADHD persistent-control group difference on nogo-P3 (Table 2).

When ADHD groups were based on parent-reports using the DSM-5 criterion, the same individuals were classified as ADHD persisters and remitters as when DSM-IV criteria were used.

### 3.6 Which objective measures are associated with the continuous ratings of self-reported ADHD symptoms and impairments at follow up in individuals with childhood ADHD?

Self-reported ADHD symptoms at follow up correlated significantly with RTV (CPT-OX & Fast Task), OE, delta, theta and alpha activity and movement count. Self-reported ADHD impairment correlated significantly only with RTV (Fast Task) and cue-P3 amplitude (Table 3). After controlling for IQ, all significant correlations remained significant, with only slight or no reduction in coefficient magnitudes (Table 4).

### 3.7 Concordant versus discordant diagnostic groups according to self- and parent-report

The concordant ADHD group (meeting ADHD criteria according to both informant reports) and discordant group (meeting ADHD criteria according to parent report only) both significantly differed from controls and not from each other on: IQ and twelve objective measures, including digit span (backward & forward), RTV (Fast Task & CPT-OX), CE, OE, No-go P3, CNV, alpha activity and movement intensity (Table 5). The pattern of results remained the same after controlling for IQ in that no measure significantly differentiated between the concordant and discordant groups. All groups significantly differed from each other on self- and parent-reported functional impairment, with the concordant group showing the highest levels of reported impairment and controls showing the lowest levels of reported impairment.

## 4 Discussion

Our follow-up study of 108 adolescents and young adults with a childhood ADHD diagnosis and 167 controls revealed that ADHD persistence and remittance based on self-report is poorly differentiated by the objective measures, as opposed to groups defined by parent-report (Cheung et al., 2015). Although individuals with persistent ADHD showed impairments relative to controls on most objective measures, the objective measures did not differentiate well between ADHD persisters and remitters. Overall, individuals with childhood ADHD rated their levels of symptoms and impairments as less severe than parents, leading to markedly different prevalence rates of ADHD depending on rater. These findings suggest that: (1) adolescents and young adults with ADHD tend to report their levels of symptoms and impairments as lower than their parents; (2) prevalence rates vary markedly according to informant source; and (3) adolescents and young adults’ reports of ADHD outcome are not as well reflected by objective cognitive, neurophysiological and movement measures as parent-reports.

Individuals with persistent ADHD showed significant impairments on nearly all objective measures, suggesting that ADHD persisters defined by self-report show similar profiles to ADHD persisters defined by parent-report. However, individuals who reported themselves as ADHD remittent showed similar profiles of underlying impairments as individuals who reported themselves as persistent. In contrast, when ADHD outcome was based on parent-reports (Cheung et al., 2015), ADHD persisters were impaired on all objective measures, while remitters did not differ from controls on any measures. ADHD remitters based on parent-reports differed from ADHD persisters but not controls on several measures, suggesting that these were markers of remission. Thus, the objective data was far better at distinguishing between persistent and remittent groups when these were based on parent-report, compared to self-report. These findings were similar when the revised DSM-5 symptom criteria for ADHD were applied to classify diagnostic status at follow-up. Furthermore, the concordant (meeting ADHD criteria according to both informant reports) and discordant (meeting ADHD criteria according to parent report only) groups significantly differed from controls on most measures and did not differ from each other on any objective measure, suggesting that self-reports of ADHD at follow-up added little value over and above parent-report alone in the association of ADHD with the objective measures studied.

The analyses on continuous measures of ADHD symptoms revealed that self- and parent-reports showed similar patterns of associations with the objective measures, suggesting a quantitative difference between self- and parent-reported symptoms, as they differed in mean severity. Self-reported impairment correlated significantly with fewer objective measures than parent-reported impairment, suggesting a qualitative difference between the informants despite the moderately strong correlation (r = 0.48, p < 0.001) between them. This suggests that individuals evaluate their level of impairment based on other factors than their parents. Further investigations into self-reported impairment and its correlates would be beneficial in order to understand on what basis young individuals estimate their levels of impairment.

It is important to acknowledge that there were notable discrepancies in results depending on which informant was used. The ADHD persistence rate based on self-reports was almost half the persistence rate based on parent-report. Furthermore, whereas several markers of remission were identified when ADHD status was based on parent-reports (Cheung et al., 2015), no markers of remission were identified using self-report. These discrepancies highlight the need for researchers to acknowledge differences in findings due to informant source used, which may explain inconsistencies in the ADHD literature across studies using different informants to measure ADHD.

Taken together with other research showing rater effects on ADHD prevalence rate at follow-up (Barkley et al., 2002) and heritability estimates (Merwood et al., 2013), further research is needed to clarify which rater is most valid. Based on the available data we would argue that parent ratings continue to be important in adolescence and young adulthood, as they appear to better reflect objective measures of impairment, as well as measures such as the heritability of ADHD. These findings may be particularly pertinent to recent publications suggesting that ADHD persistence rate in adults is very low (Agnew-Balis et al., 2016, Caye et al., 2016, Moffitt et al., 2015), as these are based on self reports.

A limitation of this study is the wide age range of the sample. Although age was controlled for in the quantitative analyses and there were no significant group differences on age, it would be important to investigate the validity of self- vs parent-report using a narrower age group, in particular individuals in their transition into young adulthood. Furthermore, only cases diagnosed with ADHD combined type in childhood were included in the sample in order to reduce heterogeneity in the sample. Thus, findings may not generalize to other presentations of ADHD. Moreover, we acknowledge that the term ‘remitters’, when based on self-reports, does not necessarily reflect a group of individuals who have remitted from self-reported ADHD, as self-reports were not obtained in childhood. Furthermore, although we found that self-report of ADHD outcome was not well reflected by objective measures, it is possible that self-report is better captured by other measures not included in our study.

In summary, this is the first study to suggest that self-report of ADHD outcome in adolescents and young adults is not as well reflected by cognitive-neurophysiological and movement data as parent-report. Our findings also demonstrate that there can be considerable inconsistencies in research findings based on the informant source used, which is important for researchers to acknowledge. For clinicians the findings suggest that during the follow-up of children with ADHD, care should be taken to continue to gather reports from multiple informants including parents.

## Contribution disclosure

Ebba Du Rietz was involved in forming the research questions, executed the statistical analyses and wrote up the report. Dr Celeste Cheung contributed to the planning of the study design, collected the data and provided support during analysis and writing of the report. Professor Jonna Kuntsi played a key role in planning the study design and research questions, and provided input for interpretation of results and the write up of the report. Professor Philip Asherson also contributed with his input regarding the research questions, interpretation of results and writing of the report. Dr Grianne McLoughlin, Professor Daniel Brandeis and Professor Banaschewski contributed by giving input on data interpretation and on the writing of the report. All authors have approved the final article.

## Role of funding source

This project was supported by generous grants from Action Medical Research and The Peter Sowerby Charitable Foundation (grant GN1777 to J Kuntsi). Initial cognitive assessments of the ADHD and control groups in childhood, and the recruitment of the control sample were supported by UK Medical Research Council grant G0300189 to J Kuntsi. Initial sample recruitment of the ADHD group was supported by NIMH grant R01MH062873 to SV Faraone. E Du Rietz is supported by a doctoral studentship from UK Medical Research Council. The funding sources had no involvement in decisions regarding study design, data collection, analysis, interpretation of data or in writing of the report.

## Conflict of interest

Professor Banaschewski has served as adviser or consultant for Bristol Myers-Squibb, Develco Pharma, Lilly, Medice, Novartis, Shire, and Vifor Pharma; he has received conference attendance support and conference support or speakers honoraria from Janssen McNeil, Lilly, Medice, Novartis, and Shire and has been involved in clinical trials conducted by Lilly and Shire. Professor Asherson has acted in an advisory role for Shire, Janssen-Cilag, Eli-Lilly and Flynn Pharma. He has received education or research grants from Shire, Janssen-Cilag and Eli-Lilly. He has given talks at educational events sponsored by the above companies. Ebba Du Rietz, Dr Celeste Cheung, Dr Grainne McLoughlin, Professor Dr Daniel Brandeis and Professor Jonna Kuntsi report no biomedical financial interests or potential conflicts of interest.

## Figures and Tables

**Table 1:** Comparisons on age, sex, IQ, digit span, cognitive, event-related potential (ERP), electroencephalogram (EEG) and actigraph measures between ADHD groups based on self-report.

	ADHD persisters (n = 48)	ADHD remitters (n = 60)	Controls (n = 167)	*F*	*df*	*p*	*Cohen's d*ʹ	*Cohen's d*ʹ *(IQ controlled)*
Mean age (SD)	18.54 (2.89)	18.34 (3.19)	17.77 (2.20)	1.94	2, 191	0.15		
Male *n (%)*	39 (81%)	54 (90%)	127 (76%)	4.07	2, 191	**0.02**		
*Cognitive measures*								
IQ	98.25 (17.06)	97.20 (13.86)	110.23 (12.15)	21.76	2, 191	**<0.01**	a = −0.58^∗∗^ b = 0.07 c = −0.80^∗∗^	
Digit span forward	9.60 (2.45)	9.38 (1.74)	10.46 (2.15)	7.46	2, 190	**<0.01**	a = −0.31^∗^ b = 0.10 c = −0.51^∗∗^	a = −0.09 b = 0.06 c = −0.17
Digit span backward	6.17 (2.37)	6.62 (2.42)	8.04 (2.61)	12.34	2, 190	**<0.01**	a = −0.61^∗∗^ b = −0.19 c = −0.51^∗∗^	a = −0.38^∗^ b = −0.27 c = −0.16
RTV (CPT-OX)	99.55 (51.97)	107.00 (60.77)	79.05 (36.96)	5.82	2, 190	**<0.01**	a = 0.34^∗^ b = −0.07 c = 0.39^∗∗^	a = 0.21 b = −0.05 c = 0.26
RTV (Fast Task)	4.77 (0.77)	4.37 (0.86)	3.76 (0.89)	28.67	2, 191	**<0.01**	a = 0.99^∗∗^ b = 0.50^∗∗^ c = 0.59^∗∗^	a = 0.80^∗∗^ b = 0.58^∗∗^ c = 0.36^∗∗^
CE (CPT-OX)	1.96 (2.44)	1.98 (2.37)	0.86 (1.33)	10.04	2, 190	**<0.01**	a = 0.40^∗∗^ b = −0.06 c = 0.52^∗∗^	a = 0.29^∗^ b = −0.04 c = 0.32^∗^
OE (CPT-OX)	2.79 (3.76)	2.01 (3.78)	0.60 (1.00)	14.00	2, 189	**<0.01**	a = 0.61^∗∗^ b = 0.34 c = 0.44^∗∗^	a = 0.52^∗∗^ b = 0.38 c = 0.22
*ERPs (CPT-OX)*								
CNV	−2.76 (1.80)	−3.21 (1.83)	−3.84 (1.86)	6.75	2, 187	<**0.01**	a = 0.47^∗∗^ b = 0.25 c = 0.28^∗^	a = 0.43^∗∗^ b = 0.25 c = 0.21
Cue P3	6.51 (0.50)	6.71 (0.56)	6.81 (0.44)	6.86	2, 187	<**0.01**	a = −0.50^∗∗^ b = −0.38 c = −0.19	a = −0.51^∗∗^ b = −0.38 c = −0.18
No-go P3	7.00 (0.46)	7.06 (0.36)	7.17 (0.38)	3.33	2, 179	**0.04**	a = −0.30^∗^ b = −0.13 c = −0.26	a = −0.23 b = −0.13 c = −0.16
*EEG frequency bands (CPT-OX)*								
Delta	1.54 (0.49)	1.58 (0.54)	1.45 (0.43)	1.58	2, 188	0.21	a = 0.16 b = −0.08 c = 0.22	a = −0.03 b = −0.07 c = 0.03
Theta	−0.15 (0.53)	−0.15 (0.55)	−0.25 (0.51)	1.60	2, 188	0.20	a = 0.19 b = −0.08 c = 0.20	a = 0.03 b = 0.01 c = 0.03
Alpha	−0.35 (0.62)	−0.41 (0.72)	−0.59 (0.61)	3.43	2, 189	**0.03**	a = 0.33^∗^ b = 0.10 c = 0.22	a = 0.26 b = 0.10 c = 0.16
Beta	−1.65 (0.69)	−1.63 (0.54)	−1.80 (0.57)	2.24	2, 189	0.11	a = 0.18 b = −0.04 c = 0.27	a = 0.10 b = −0.03 c = 0.15
*Actigraph movement*								
Mean intensity	1.20 (0.74)	1.03 (0.69)	0.77 (0.55)	7.83	2, 168	<**0.01**	a = 0.54^∗∗^ b = 0.25 c = 0.34^∗^	a = 0.45^∗∗^ b = 0.26 c = 0.25
Mean count	0.05 (0.04)	0.04 (0.04)	0.03 (0.06)	5.82	2, 141	<**0.01**	a = 0.42^∗∗^ b = 0.26 c = 0.27	a = 0.33^∗^ b = 0.25 c = 0.19

RTV, reaction time variability; CE, commission errors; OE, omission errors; CNV, continuous negative variation *p-value < 0.05, ** p-value < 0.01. P-values presented in bold are significant.

a ADHD persisters vs controls.

b ADHD persisters vs ADHD remitters.

c ADHD remitters vs controls.

**Table 2:** Comparisons on age, sex, IQ, digit span, cognitive, event-related potential (ERP), electroencephalogram (EEG) and actigraph measure between ADHD groups based on self-report using DSM-5 criteria.

	ADHD persisters (n = 51)	ADHD remitters (n = 57)	Controls (n = 167)	*F*	*df*	*p*	*Cohen's d*ʹ
Mean age (SD)	18.55 (2.81)	18.32 (3.27)	17.77 (2.20)	2.03	2, 191	0.13	
Male *n (%)*	42 (82%)	51 (89%)	127 (76%)	3.59	2, 191	**0.03**	
*Cognitive measures*							
IQ	98.29 (16.58)	97.11 (14.18)	110.23 (12.15)	21.41	2, 191	<**0.01**	a = −0.61^∗∗^ b = 0.08 c = −0.79^∗∗^
Digit span forward	9.64 (2.39)	9.33 (1.76)	10.46 (2.15)	7.72	2, 190	<**0.01**	a = −0.30^∗^ b = 0.15 c = −0.51^∗∗^
Digit span backward	6.18 (2.31)	6.63 (2.45)	8.04 (2.61)	12.50	2, 190	<**0.01**	a = −0.62^∗∗^ b = −0.19 c = −0.48^∗∗^
RTV (CPT-OX)	96.62 (51.76)	109.96 (60.88)	79.05 (36.96)	6.08	2, 190	<**0.01**	a = 0.29^∗^ b = −0.19 c = 0.43^∗∗^
RTV (Fast Task)	4.78 (0.89)	4.39 (0.86)	3.76 (0.89)	26.43	2, 191	<**0.01**	a = 0.94^∗∗^ b = 0.40^∗^ c = 0.59^∗∗^
CE (CPT-OX)	1.86 (2.63)	2.07 (2.40)	0.86 (1.33)	10.33	2, 190	<**0.01**	a = 0.37^∗∗^ b = −0.15 c = 0.54^∗∗^
OE (CPT-OX)	2.60 (3.72)	2.11 (3.86)	0.60 (1.00)	13.59	2, 189	<**0.01**	a = 0.60^∗∗^ b = 0.26 c = 0.45^∗∗^
*ERPs (CPT-OX)*							
CNV	−2.90 (1.91)	−3.11 (1.74)	−3.84 (1.86)	5.92	2, 187	<**0.01**	a = 0.40^∗∗^ b = 0.11 c = 0.33^∗^
Cue P3	6.55 (0.51)	6.69 (0.56)	6.81 (0.44)	6.36	2, 187	<**0.01**	a = −0.45^∗∗^ b = −0.22 c = −0.26
No-go P3	7.00 (0.50)	6.99 (0.45)	7.17 (0.38)	3.33	2, 179	**0.04**	a = −0.26 b = −0.03 c = −0.29^∗^
*EEG frequency bands (CPT-OX)*							
Delta	1.54 (0.54)	1.61 (0.54)	1.45 (0.43)	1.88	2, 188	0.16	a = 0.11 b = −0.20 c = 0.26
Theta	−0.17 (0.53)	−0.09 (0.61)	−0.25 (0.51)	1.66	2, 188	0.19	a = 0.16 b = −0.10 c = 0.23
Alpha	−0.37 (0.61)	−0.40 (0.73)	−0.59 (0.61)	3.22	2, 189	**0.04**	a = 0.31^∗^ b = 0.04 c = 0.23
Beta	−1.68 (0.68)	−1.60 (0.54)	−1.80 (0.57)	2.69	2, 189	0.07	a = 0.15 b = −0.13 c = 0.31^∗^
*Actigraph movement*							
Mean intensity	1.15 (0.75)	1.06 (0.68)	0.77 (0.55)	7.44	2, 168	<**0.01**	a = 0.49^∗∗^ b = 0.14 c = 0.37^∗^
Mean count	0.05 (0.04)	0.04 (0.04)	0.03 (0.06)	5.71	2, 141	<**0.01**	a = 0.48^∗∗^ b = 0.25 c = 0.32^∗^

RTV, reaction time variability; CE, commission errors; OE, omission errors; CNV, continuous negative variation * p-value < 0.05, ** p-value < 0.01. P-values presented in bold are significant.

a ADHD persisters vs controls.

b ADHD persisters vs ADHD remitters.

c ADHD remitters vs controls.

**Table 3:** Pearson correlations (two-tailed) of IQ, digit span, cognitive, event-related potential (ERP), electroencephalogram (EEG) and actigraph measures with self-reported symptoms and impairment in individuals with childhood ADHD (N = 108).

	ADHD symptoms		Impairment	
	*r*	*p*	*r*	*p*
IQ	−0.12	0.21	0.02	0.81
Digit span forward	0.02	0.83	−0.02	0.83
Digit span backward	−0.07	0.45	−0.01	0.92
RTV (CPT-OX)	**0.23**	**0.02**	0.02	0.82
RTV (Fast Task)	**0.33**	<**0.01**	**0.21**	**0.03**
Commission errors	−0.01	0.94	0.04	0.70
Omission errors	**0.24**	**0.01**	0.18	0.07
CNV	0.18	0.07	0.06	0.53
Cue P3	−0.14	0.15	**−0.19**	**0.05**
No Go P3	−0.01	0.93	0.03	0.75
Delta	**0.21**	**0.03**	−0.01	0.89
Theta	**0.21**	**0.04**	0.01	0.91
Alpha	**0.19**	**0.04**	0.14	0.16
Beta	0.09	0.37	0.09	0.35
Movement intensity	0.18	0.09	0.06	0.60
Movement count	**0.26**	**0.03**	0.18	0.15

RTV, reaction time variability; CE, commission errors; OE, omission errors; CNV, continuous negative variation. Significant values are presented in bold.

**Table 4:** Pearson correlations of IQ, digit span, cognitive, event-related potential (ERP), electroencephalogram (EEG) and actigraph measures with self-reported symptoms and clinical impairment in individuals with childhood ADHD (N = 108); controlling for IQ.

	ADHD symptoms		Impairment	
	*r*	*p*	*r*	*p*
Digit span forward	0.07	0.49	−0.03	0.76
Digit span backward	−0.03	0.75	−0.02	0.84
RTV (CPT-OX)	**0.21**	**0.03**	0.02	0.83
RTV (Fast Task)	**0.31**	<**0.01**	**0.23**	**0.03**
Commission errors	−0.04	0.69	0.05	0.64
Omission errors	**0.22**	**0.02**	0.19	0.06
CNV	0.17	0.09	0.06	0.62
Cue P3	−0.13	0.20	**−0.20**	**0.05**
No Go P3	<0.01	0.96	0.03	0.75
Delta	0.19	0.06	−0.01	0.89
Theta	**0.19**	**0.05**	0.01	0.89
Alpha	**0.19**	**0.05**	0.14	0.16
Beta	0.09	0.35	0.09	0.35
Movement intensity	0.17	0.11	0.05	0.64
Movement count	**0.26**	**0.04**	0.17	0.19

RTV, reaction time variability; CE, commission errors; OE, omission errors; CNV, continuous negative variation. Significant values are presented in bold.

**Table 5:** Comparison on objective measures and reports of impairments between ADHD concordant and discordant groups and controls.

	Controls (n = 167)	Concordant ADHD group (n = 43)	Discordant ADHD group (n = 42) (only parent-reported ADHD)
Age	17.77 (2.20)	18.45 (2.90)	18.16 (3.23)
Male n (%)	127^c^	34	36^a^
IQ	110.23 (12.15)^b^^,^^c^	97.35 (17.23)^a^	94.21 (12.96)^a^
DSF	10.46 (2.15)^b^^,^^c^	9.26 (2.36)^a^	9.33 (1.65)^a^
DSB	8.04 (2.61)^b^^,^^c^	5.98 (2.36)^a^	6.48 (2.45)^a^
RTV (CPT-OX)	79.05 (36.96)^b^^,^^c^	99.02 (47.56)^a^	122.81 (63.26)^a^
RTV (Fast Task)	3.76 (0.89)^b^^,^^c^	4.86 (0.90)^a^	4.59 (0.77)^a^
CE	0.86 (1.33)^b^^,^^c^	2.05 (2.74)^a^	2.17 (2.47)^a^
OE	0.60 (1.00)^b^^,^^c^	2.95 (3.87)^a^	2.64 (4.36)^a^
Cue P3	6.81 (0.44)^b^	6.52 (0.52)^a^	6.63 (0.56)
No-go P3	7.17 (0.38)^b^^,^^c^	6.99 (0.51)^a^	6.94 (0.48)^a^
CNV	−3.84 (1.86)^b^^,^^c^	−2.80 (1.87)^a^	−2.81 (1.75)^a^
Delta	1.45 (0.43)^c^	1.59 (0.54)	1.68 (0.56)^a^
Theta	−0.25 (0.51)^c^	−0.14 (0.55)	−0.02 (0.65)^a^
Alpha	−0.59 (0.61)^b^^,^^c^	−0.37 (0.64)^a^	−0.33 (0.77)^a^
Beta	−1.80 (0.57)^c^	−1.67 (0.66)	−1.56 (0.54)^a^
Actigraph intensity	0.77 (0.55)^b^^,^^c^	1.27 (0.76)^a^	1.11 (0.72)^a^
Actigraph count	0.03 (0.06)^b^	0.05 (0.04)^a^	0.05 (0.04)
Parent-reported impairment	2.73 (3.32)^b^^,^^c^	17.44 (4.97)^a^^,^^c^	15.26 (5.49)^a^^,^^b^
Self-reported impairment	3.29 (3.14)^b^^,^^c^	15.56 (4.46)^a^^,^^c^	8.08 (4.20)^a^^,^^b^

Concordant group: meeting ADHD criteria according to both self- and parent-report.

Discordant group: meeting ADHD criteria according to parent-report only.

a Significantly (p < 0.05) different from controls.

b Significantly (p < 0.05) different from concordant ADHD group.

c Significantly (p < 0.05) different from discordant ADHD group.
